# Predictive and Prognostic Impact of Blood-Based Inflammatory Biomarkers in Patients with Gastroenteropancreatic Neuroendocrine Tumors Commencing Peptide Receptor Radionuclide Therapy

**DOI:** 10.3390/diagnostics11030504

**Published:** 2021-03-12

**Authors:** Fiona Ohlendorf, Rudolf A. Werner, Christoph Henkenberens, Tobias L. Ross, Hans Christiansen, Frank M. Bengel, Thorsten Derlin

**Affiliations:** 1 Department of Nuclear Medicine, Hannover Medical School, 30625 Hannover, Germany; Fiona.Ohlendorf@stud.mh-hannover.de (F.O.); Werner.Rudolf@mh-hannover.de (R.A.W.); Ross.Tobias@mh-hannover.de (T.L.R.); Bengel.Frank@mh-hannover.de (F.M.B.); 2 Department of Radiation Oncology, Hannover Medical School, 30625 Hannover, Germany; Henkenberens.Christoph@mh-hannover.de (C.H.); Christiansen.Hans@mh-hannover.de (H.C.)

**Keywords:** neuroendocrine tumors, inflammation, prognosis, somatostatin receptor, PET/CT, PRRT

## Abstract

Tumor microenvironment inflammation contributes to the proliferation and survival of malignant cells, angiogenesis, metastasis, subversion of adaptive immunity, and reduced treatment response. We aimed to evaluate the early predictive and prognostic significance of markers of systemic inflammation in patients receiving somatostatin-receptor targeted peptide receptor radionuclide therapy (PRRT). This retrospective observational cohort study included 33 patients with advanced gastro-entero-pancreatic neuroendocrine tumors (GEP-NETs) treated with PRRT. Pretreatment blood-based inflammatory biomarkers, e.g., C-reactive protein levels (CRP), white blood cell count (WBC), and absolute neutrophil count (ANC), were documented and inflammation indexes, e.g., neutrophil-lymphocyte ratio (NLR) and Platelet × CRP multiplier (PCM), were calculated. Tumor burden was determined using [^68^Ga]Ga-DOTA-TATE PET/CT before enrollment and every 2 cycles thereafter until progression. Therapy response was assessed using RECIST 1.1, including its volumetric modification. Inflammatory biomarkers and inflammatory indexes demonstrated marked heterogeneity among patients, and were significantly higher in non-responders (e.g., CRP (*p* < 0.001), ANC (*p* = 0.002), and PCM (*p* < 0.001)). Change in whole-body tumor burden after two cycles of PRRT was significantly associated with CRP (*p* = 0.0157) and NLR (*p* = 0.0040) in multivariate regression analysis. A cut-off of 2.5 mg/L for CRP (AUC = 0.84, *p* = 0.001) revealed a significant outcome difference between patients with adversely high vs. low CRP (median PFS 508 days vs. not yet reached (HR = 4.52; 95% CI, 1.27 to 16.18; *p* = 0.02)). Tumor-driven systemic inflammatory networks may be associated with treatment response, change in tumor burden, and prognosis in patients with GEP-NETs receiving PRRT.

## 1. Introduction

Peptide receptor radionuclide therapy (PRRT) with radioactively-labeled 1, 4, 7, 10-tetraazacyclododecane-N,N′,N″,N‴-tetraaceticacid-d-Phe(1)-Tyr(3)-octreotate (DOTA-TATE) has been approved for the treatment of unresectable or metastatic, progressive, well differentiated (G1 and G2), somatostatin receptor (SSR)-positive gastroenteropancreatic neuroendocrine tumours (GEP-NETs) in adults [1,2,3]. PRRT with ^177^Lutetium-[^177^Lu]Lu-DOTA-TATE has been shown to induce objective radiological response in up to 18% of patients, and to significantly improve progression-free survival in advanced midgut NETs [1]. However, a substantial number of patients will not respond to PRRT, or eventually progress, underlining an unmet clinical need for better prediction of the anti-tumor effect of this therapy, and guidance for therapeutic decision making. The role of cancer-related inflammation has recently gained increased interest. Chronic inflammatory conditions increase cancer risk [4,5], but genetic alterations may also generate an inflammatory environment due to the production of inflammatory mediators including cytokines and chemokines [6,7,8]. Ultimately, these recruit and activate various leukocytes, most notably cells of the myelomonocytic lineage [9]. In the tumor microenvironment, smoldering inflammation contributes to the proliferation and survival of malignant cells, angiogenesis, metastasis, subversion of adaptive immunity, reduced response to hormones, and chemotherapeutic agents [8,10]. Consistently, both the immune infiltration in human tumors [11] and various blood-based inflammatory biomarkers have demonstrated prognostic significance in various types of cancer [12,13,14]. Recently, Zou et al. have investigated the role of systemic inflammation-based markers in patients with inoperable advanced or metastatic NETs, and suggested that the high-sensitivity inflammation-based prognostic index (HSPI) was an independent predictor of shorter overall survival [15], underlining the potential prognostic significance of cancer-related inflammation in NETs. In the setting of external beam radiation therapy, tumor-related pretreatment leukocytosis and neutrophilia have been associated with resistance to radiotherapy, immune suppression, and promotion of metastasis [16,17,18]. Furthermore, both pre-treatment and treatment-related lymphocytopenia has been associated with poor outcome [18,19]. To the best of our knowledge, data on the clinical relevance of systemic inflammation in NETs in the context of PRRT are very limited. Based on previous data about cancer-related inflammation and experience with external radiation therapy, we speculated that blood-based inflammatory biomarkers may also have both predictive and prognostic significance in the context of internal theranostic radionuclide therapy. We tested this hypothesis in a cohort of patients with gastroenteropancreatic NETs undergoing PRRT, and analyzed the relationship between systemic inflammatory biomarkers and outcome.

## 2. Materials and Methods

### 2.1. Study Cohort 

A total of 33 patients (19 women, 14 men; mean age, 63.4 ± 11.6 years (y) (range, 39–81 y)) with advanced gastroenteropancreatic NETs who underwent at least two cycles of PRRT between May 2012 and September 2019 were included in this retrospective study. PRRT was performed according to the joint International Atomic Energy Agency (IAEA; Vienna, Austria), European Association of Nuclear Medicine (EANM; Vienna, Austria), and Society of Nuclear Medicine and Molecular Imaging (SNMMI; Reston, VA, USA) practical guidance in accordance with the Rotterdam protocol as published [20]. Patients were treated until progression (*n* = 8) or until occurrence of dose-limiting toxicity (*n* = 2), or if no therapeutic effect could be achieved (*n* = 4) or until they refused further treatment (*n* = 0) or until a treatment pause was indicated due to the patient’s medical condition (*n* = 2) or due to stabilized disease status and freedom of symptoms (*n* = 7). PRRT treatment was ongoing at the time of analysis in the remaining patients. A panel of routine blood-based parameters was determined before each treatment cycle, including inflammatory markers such as C-reactive protein levels (CRP), white blood cell count (WBC), absolute neutrophil and absolute lymphocyte count, platelet count, and platelet volume. In addition, a panel of different scores quantifying systemic inflammation was calculated, e.g., the three-point scale high-sensitivity inflammation-based prognostic index (HSPI) [15]. For the HSPI, patients with CRP levels < 3 mg/L and a WBC count < 11 × 10^9^/L were classified with a HSPI score of 0. Patients with one elevated parameter, either CRP ≥ 3 mg/L and WBC < 11 × 10^9^/L or CRP < 3 mg/L and WBC ≥ 11 × 10^9^/L, were rated with a HSPI score of 1. With both parameters being elevated (CRP ≥ 3 mg/L and WBC ≥ 11 × 10^9^/L), a HSPI score of 2 was reached. Furthermore, the neutrophil-lymphocyte ratio (NLR) [21], the platelet × CRP multiplier (PCM) value, the platelet-lymphocyte ratio (PLR), and the CRP-albumin ratio (CRP/Alb ratio) were calculated. Besides, a panel of other laboratory values was assessed including chromogranin A (CgA), standard hematology, liver enzymes, alkaline phosphatase (ALP), and lactate dehydrogenase (LDH). All patients gave written informed consent for the diagnostic and therapeutic procedures as well as for the retrospective scientific analysis of their data. The SSR ligands were administered in compliance with the Declaration of Helsinki, §37 and the German Medicinal Products Act, AMG §13.2b. The institutional review board approved this retrospective study (No. 8017_BO_S_2018).

### 2.2. GMP-Compliant Preparation of the SSR-Targeting Ligands 

Lutetium-177 was purchased from itg (Isotope Technologies Garching GmbH, Garching, Germany) as GMP-certified [^177^Lu]LuCl3 in 0.04M HCl-solution (EndolucinBetaTM, 40 GBq/mL) in no carrier added quality. The precursor DOTA-TATE was obtained from ABX (Radeberg, Germany) in GMP quality. The radiosynthesis was performed on a Gaia/Luna GMP automated radiosynthesizer (Elysia-raytest GmbH, Straubenhardt, Germany) using a sterile, single-use cassette and reagent kit (ABX, Radeberg, Germany). Per patient dose, 150 µg of DOTA-TATE precursor was dissolved in 800 µL of buffer solution (gentisic acid/sodium ascorbate/HCl). Between 7.0–9.0 GBq [^177^Lu]LuCl3 per patient was provided in the sterile, rubber sealed delivery vial (10 mL), which served as a reaction vessel in the automated process. The 177Lu-labelling step was conducted at 95 °C for 30 min. The product solution was transferred into a product vial via a sterile filter and diluted by 10–15 mL 0.9% NaCl. Patient doses were calculated and dispensed into 50-mL syringes with an addition of 0.9% NaCl by a self-designed automated dispensing system. The radiosynthesis automate and the dispensing system are both housed in a laminar air flow class-A glovebox under controlled conditions. RadioHPLC as the primary quality control was performed on a Merck HPLC system equipped with two L-7100 pumps, a L-7200 autosampler, a L-7400 UV/Vis detector, a D-7000 interface d-line and a GABI radiodetector (Elysia-raytest, Straubenhardt, Germany), and a Gemini C18, 5 µm, 100 Å column (250 × 4.6 mm) (Phenomenex, Aschaffenburg, Germany). An eluent phosphate buffer (pH 2) and acetonitrile was used in a gradient system at a flow of 0.6 mL/min. Production batches were further tested for pH, sterility, endotoxins, and radionuclide purity (gamma spectroscopy). [^177^Lu]Lu-DOTA-TATE was always of flawless quality with a radiochemical purity of ≥97% and a peptide content of 18.5–19.5 µg/GBq.

A total of 10/33 patients received PRRT using the SSR ligand DOTA-TOC (29 (16%) of cycles) [22,23].

### 2.3. PET/CT Image Analysis

For assessment of disease extent, patients underwent an initial [^68^Ga]Ga-DOTA-TATE PET/CT before commencing PRRT as described previously [24], and after every two cycles of PRRT. The analysis of the PET/CT images was performed using a dedicated workstation equipped with a commercial software package (syngo.via; V10B, Siemens Healthineers, Erlangen, Germany), providing a simultaneous and fused review of PET and CT data. All lesions suggestive for metastatic disease were noted, and their localization (e.g., lymph node metastases, bone metastases, and hepatic metastases) was recorded and the number of detected metastases per patient was recorded. The somatostatin receptor-derived tumor volume (SSR-TV) and total lesion SSR expression (TL-SSR, defined as mean standardized uptake value x SSR-TV) was measured for each lesion using a three-dimensional segmentation and computerized volumetric technique to create a three-dimensional isocontour volume-of-interest (VOI) including all voxels above 40% of the maximum, as described previously [24]. The whole-body tumor burden (wb-SSR-TV) was calculated by summing SSR-TV measurements of all lesions in each patient.

### 2.4. Assessment of Treatment Response and Clinical Endpoints

Treatment response was evaluated using [^68^Ga]Ga-DOTA-TATE PET/CT according to RECIST 1.1 criteria, adapted to volumetric measurements [24,25,26]. Patients underwent a baseline PET before first PRRT, followed by a PET/CT after two cycles. In patients already having received PRRT, PET/CT was performed following every two cycles. Non-responders were defined as patients with progressive disease, i.e., appearance of new lesions or an increase in tumor burden greater or equal to 73%. A partial remission was defined as a decrease in tumor burden greater or equal to 63%. Both partial remission and stable disease during follow-up were considered as treatment response. Progression-free survival was defined as the time period between the date of the initial [^68^Ga]Ga-DOTA-TATE PET/CT imaging before the first PRRT cycle and the date of the first [^68^Ga]Ga-DOTA-TATE PET/CT imaging with signs of disease progression as listed above.

### 2.5. Statistical Analysis

Categorical variables are presented with absolute and relative frequencies. Continuous variables are expressed as mean ± standard deviation (SD) and range. Due to a lack of consistent normal distribution among all baseline laboratory parameters, non-parametric tests were used throughout statistical analysis. Laboratory data of responders and non-responders was compared using a Mann–Whitney-U-test for independent continuous variables. Chi-square test was used to analyze differences in discrete variables between responders and non-responders. For univariate analyses, binary logistic regression was performed to identify factors predictive of treatment response and linear regression was used for metric variables, such as change in wb-SSR-TV. All parameters showing a significant result in the univariate analysis and all inflammatory markers were included in a multivariate analysis using an eligible regression model. Receiver operating characteristics (ROC) analysis was performed to identify optimal cut-off values for inflammatory parameters. The Kaplan–Meier method was used to create survival curves for PFS. Cox regression was performed using nominal variables. Cut-offs for the transcription into nominal variables were determined by ROC analysis or, if no optimal cut-off could be identified, the median of each variable was used as cut-off value. A *p*-value ≤ 0.05 was considered a significant result. Statistical analysis was performed using IBM SPSS Statistics 25 (IBM, Armonk, NY, USA) for macOS Mojave and GraphPad Prism v8.0 (GraphPad Software, San Diego, CA, USA) for Windows.

## 3. Results

### 3.1. Patient Characteristics and Extent of Disease

A total of 33 patients (Table 1) with GEP-NETs who underwent a total of 181 PRRT cycles (5.5 ± 2.3 (range, 2–13) per patient and with a mean administered activity of 7.4 GBq per cycle) were analyzed. During the follow-up period of 30 ± 20 months (range, 9–84 months), 16/33 (48.5%) patients progressed, while 17/33 (51.5%) patients showed a response to PRRT. Mean progression-free survival was 22 ± 16 months (range, 3–71 months). 3/33 (9%) patients died from their tumor during follow-up (mean overall survival 814 ± 348 days (range, 462–1157 days). 

When analyzing the extent of disease on [^68^Ga]Ga-DOTA-TATE PET/CT, a total of 852 tumor lesions were identified. A total of 103 (12.1%) lymph node metastases were found in 24/33 (72.7%) patients, 407 (47.8%) bone metastases were detected in 20/33 (60.6%) patients, 283 (33.2%) liver metastases were found in 31/33 (93.9%) patients, and 52 (6.1%) other metastases were identified in 16 (48.5%) patients. In 7/33 (21.2%) patients, a primary was still present (7/852, 0.8%). 

### 3.2. Baseline Markers of Systemic Inflammation Are Elevated in Non-Responders to PRRT

**Figure 1:**
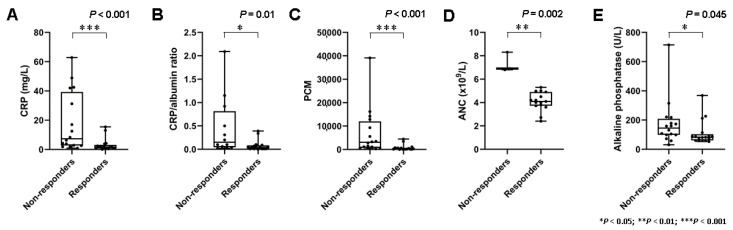
Markers of systemic inflammation and treatment response. (**A**) Box plots demonstrating significantly higher serum levels of C reactive protein levels (CRP) (*p* < 0.001), (**B**) CRP/albumin ratio (*p* = 0.01), (**C**) platelet × CRP multiplier (PCM) (*p* < 0.001), and (**D**) absolute neutrophile count (ANC) (*p* = 0.002) in non-responders. (**E**) Serum levels of alkaline phosphatase (ALP) (*p* = 0.045) were also associated with response, albeit less clearly.

A total of 16 patients did not respond, and 7 (44%) of these demonstrated early progressive disease with the development of new metastases following two cycles of PRRT. Baseline blood-based markers of systemic inflammation demonstrated marked interindividual heterogeneity (e.g., CRP (range, 0.08 mg/L to 62.8 mg/L)). On a per-group basis, non-responders had significantly higher baseline markers of systemic inflammation such as CRP (3.0 ± 4.4 mg/L vs. 18.1 ± 20.4 mg/L; *p* < 0.001), CRP/albumin ratio (0.079 ± 0.115 vs. 0.46 ± 0.63; *p* = 0.01), ANC (4.1 ± 0.8 × 10^9^/L vs. 7.3 ± 0.8 × 10^9^/L; *p* = 0.002), and the PCM (776 ± 1241 vs. 7011 ± 10092; *p* < 0.001) (Figure 1). 

In line with these results, HSPI scores (11/16 with HSPI score of 1 in the group of non-responders vs. 4/17 with HSPI score of 1 in the group of responders; *p* = 0.015) were significantly higher in non-responders. Furthermore, there was a significant difference in serum levels of ALP (111 ± 83 U/L vs. 178 ± 160 U/L; *p* = 0.045). Detailed data on baseline blood-based parameters in non-responders and responders are shown in Table 2.

### 3.3. Early Change in Tumor Burden Is Associated with Markers of Systemic Inflammation 

**Figure 2:**
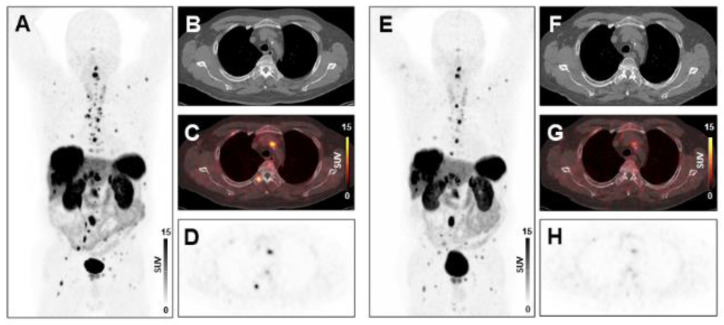
Treatment response to peptide receptor radionuclide therapy (PRRT) in a patient with low systemic inflammation at baseline. Maximum-intensity-projection (MIP) image (**A**) and transversal PET/CT images (**B**–**D**) of baseline [^68^Ga]Ga-DOTA-TATE PET/CT showing osseous, hepatic, and lymph node metastases in a 66-years-old male gastro-entero-pancreatic neuroendocrine tumor (GEP-NET) patient. MIP image (**E**) and transversal PET/CT images (**F**–**H**) at follow-up after two cycles of PRRT demonstrating decreasing tracer uptake or disappearance of lesions (SSR-TV decreased from 496 cm^3^ to 277 cm^3^, consistent with stable disease). The baseline CRP level was 1.6 mg/L, ANC was 4.0 × 10^9^/L, the PCM was 227.2, and the HSPI was 0, evidencing low systemic inflammation.

**Figure 3:**
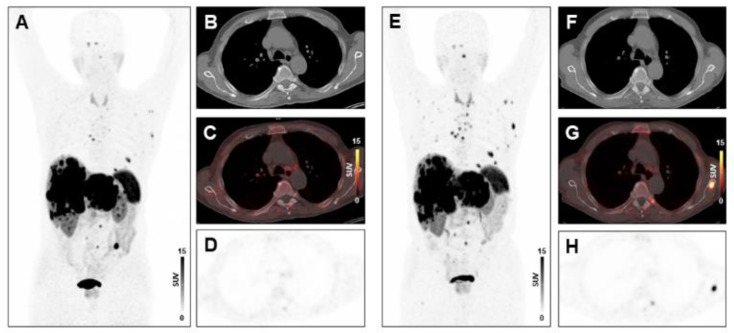
Absence of treatment response to PRRT in a patient with high systemic inflammation at baseline. MIP image (**A**) and transversal PET/CT images (**B**–**D**) of baseline [^68^Ga]Ga-DOTA-TATE-PET/CT showing osseous and hepatic metastases in a 76-years-old male GEP-NET patient. MIP image (**E**) and transversal PET/CT images (**F**–**H**) at follow-up after two cycles of PRRT demonstrating progression with new osseous lesions (SSR-TV decreased mildly from 797.26 cm^3^ to 734.09 cm^3^, however new lesions appeared, consistent with progressive disease). The baseline CRP level was 31.2 mg/L, the PCM was 14,196, and the HSPI 1, evidencing high systemic inflammation.

Following two cycles of PRRT, tumor burden changed by −13.7% ± 49.8% (range, −87.4% to 147.3%) in the total study population. In multivariate regression analysis, CRP (*p* = 0.0157) and NLR (*p* = 0.0040) were significant predictors of change in tumor burden following two cycles of PRRT. In univariate analysis, other inflammatory markers including leukocyte count (*p* = 0.0033), absolute neutrophil count (*p* = 0.0193), and the PLR (*p* = 0.0349) were also associated with change in tumor burden (Supplementary Table S1). Example cases are shown in Figure 2 and Figure 3. 

### 3.4. Baseline Markers of Systemic Inflammation Are Associated with PFS

**Figure 4:**
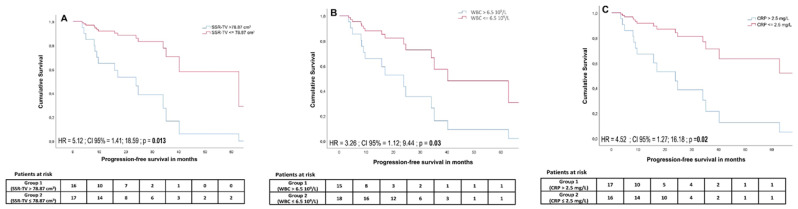
Cumulative survival curves for somatostatin receptor imaging-derived tumor volume (SSR TV) (**A**), white blood cell count (WBC) (**B**), and CRP (**C**) cut-offs estimated using a Cox proportional hazards regression model. High tumor volume, elevated white blood cell count, and elevated CRP levels were associated with significantly lower progression-free survival.

A cut-off of >2.5 mg/L for CRP was defined by receiver-operating characteristics (Area under the curve (AUC) = 0.84, *p* = 0.001) and revealed a significant outcome difference between patients with adversely high vs. low CRP (median PFS 508 days vs. not yet reached (HR = 4.52; 95% CI, 1.27 to 16.18; *p* = 0.02)). In univariate analysis, PFS was significantly associated with a CRP level ≤2.5 mg/L (HR = 4.52, 95% CI, 1.27 to 16.18; *p* = 0.02), WBC ≤ 6.5 × 10^9^/L (HR = 3.26, 95% CI, 1.12 to 9.44; *p* = 0.03), wb-SSR-TV ≤ 78.87 cm^3^ (HR = 5.12, 95% CI, 1.41 to 18.59; *p* = 0.013), PCM ≤910.45 (HR = 3.98, 95% CI, 1.25 to 12.68; *p* = 0.019), and CgA ≤ 386 μg/L (HR = 9.99, 95% CI, 1.22 to 81.93; *p* = 0.032) (Figure 4). In multivariate analysis, the cut-offs for CRP (HR = 226.33, 95% CI, 3.47 to 14768.04; *p* = 0.011), WBC (HR = 20.26, 95% CI, 1.91 to 214.36; *p* = 0.012), and wb-SSR-TV (HR = 26.67, 95% CI, 1.77 to 401.25; *p* = 0.018) remained significant. 

Detailed data on predictive parameters for PFS are shown in Table 3. A total of 3/33 (9.1%) died during the follow-up period after showing progressive disease. Both univariate and multivariate analyses did not reveal a significant association between OS and baseline markers of systemic inflammation.

## 4. Discussion

In this study, we evaluated the predictive and prognostic significance of blood-based inflammatory parameters in patients with metastatic GEP-NETs undergoing PRRT. We found that non-responders had significantly higher baseline inflammatory markers. Moreover, inflammatory markers were associated with the extent of tumor burden reduction following two cycles of PRRT, and demonstrated prognostic value.

Metastatic NETs remain challenging to treat. PRRT represents a promising therapeutic option, but it seems effective in only a subgroup of patients, creating an as yet unmet need for techniques to predict response to treatment [27]. Analysis of the SSR expression by SSR-ligand PET or conventional scintigraphic imaging is regarded as a crucial pretherapeutic requirement before PRRT, providing information about both the presence of the target and the extent of tumor burden. However, response prediction on an individual basis using SUVs is not reliably possible [28], particularly in case of SSR ligand PET [29,30,31], indicating the need for other predictors. Similarly, the proliferation index Ki-67 has some prognostic and predictive utility [32], but its usefulness is limited by tumor heterogeneity, inter-observer variability, and lack of concordance between primary tumors at the time of initial diagnosis and pre-treated metastases at PRRT [33].

Recently, the prognostic utility of markers of systemic inflammatory response has gained increased interest. In patients with pancreatic NETs (PNETs), a NLR > 2.3 (HR 2.53, 95% CI 1.05–6.08, *p* = 0.038) at baseline has been shown to be an independent predictor of disease progression, and a PLR > 160.9 (HR 5.86, 95% CI 1.27–27.08, *p* = 0.023) was independently associated with worse PFS on multivariable analysis among patients who did not undergo surgery [34]. In another study, Wiese and colleagues demonstrated that median overall survival was significantly short for patients with elevated CRP levels compared to patients with normal CRP levels (1093 days vs. 6859 days) [35]. In a large study including 620 patients, low preoperative NLRs were associated with significantly improved overall survival (*p* < 0.01) and recurrence-free survival (*p* < 0.01) in patients with resectable PNETs [36]. 

Only limited data is available regarding the significance of systemic inflammation in patients undergoing PRRT. Black and colleagues have found that an elevated inflammation-based index (IBI), derived from serum C-reactive protein and albumin levels, was associated with inferior PFS (HR, 14.2; 95% CI, 5.25 to 38.5; *p* < 0.001) and OS (*p* < 0.001), and their multivariate analysis confirmed an independent association between IBI and PFS (*p* = 0.001) in patients receiving [^177^Lu]Lu-DOTA-TATE [27]. Another study evaluating the relevance of systemic inflammation in patients receiving [^90^Y]Y-DOTATOC found that elevated baseline IBI was associated with worse OS (HR, 3.90; *p* = 0.001), and multivariate analysis corroborated an independent association between OS and IBI (*p* = 0.015) [37]. We further expanded on these findings and demonstrated that inflammatory biomarkers may also be predictive of therapy response, finding a clear association between markers and change in tumor burden after two cycles of PRRT with [^177^Lu]Lu-DOTA-TATE. In a preclinical study, Wu and colleagues highlighted the immunomodulatory effects of PRRT in the murine xenograft model of human NETs, demonstrating that PRRT causes increased infiltration of CD86^+^ antigen presenting cells and natural killer cells into tumor tissue [38]. Taken together, these studies further support our findings, and also indicate the dynamic and complex relationship between pre-existing systemic inflammation and immunomodulatory effects of the therapy itself.

Other groups have evaluated other biomarkers for response prediction. Bodei and colleagues have demonstrated the usefulness of the PRRT predictive quotient (PPQ) which integrates NET transcript expression in blood with tumor grade. Analyzing the whole-blood expression signature of four growth factor-related genes (ARAF, BRAF, KRAS, and RAF-1) and four genes involved in metabolism (ATP6V1H, OAZ2, PANK2, and PLD3), combined with tumor grade, they were able to predict the efficacy of PRRT with an accuracy of 95% [39]. However, the precise source of the transcript expression in whole-blood samples cannot be determined and may be associated with inflammatory cells that contribute a considerable fraction of blood samples. It would be desirable to further elucidate the contribution of inflammatory cells to these genomic signatures, and to also refine the PPQ using inflammation markers to even better predict treatment efficacy.

As already demonstrated by other groups, the tumor marker CgA has limited usefulness for prediction of response to PRRT. It was predictive in 49% but the efficacy in individual treatment cohorts ranged between 18 and 100% in the study by Bodei et al. [39]. Baseline CgA levels > 600 ng/mL have been shown to constitute a risk factor for early progression in patients with bone metastases in a retrospective study [40], but have limited usefulness for response prediction. Consistently, Black and colleagues also found no significant association between CgA levels and response (*p* = 0.187) within their cohort [27]. Indeed, CgA was not predictive of change in tumor burden in our cohort, confirming the need for better predictors. By contrast, the presence of positive lesions on [^18^F]F-fluorodeoxyglucose (FDG) PET has been found to represent an independent prognostic factor in patients with NETs treated with PRRT. Metabolic imaging was not performed in this cohort, but could provide additional information.

Some limitations of this study should be acknowledged. First, the sample size, the retrospective nature of this study, and the heterogeneity of the study population come along with inherent limitations. However, given the low incidence of NETs and the even lower number of patients receiving PRRT, this limitation applies to many studies in the field. Second, a small number of patients received PRRT with the ligand [^177^Lu]Lu-DOTATOC. Although no relevant influence on the reported results is to be expected, this may add some minor tracer-related variability in the assessment among patients. Regarding the performance of both SSR-ligands in PET, Velikyan and colleagues found no significant differences in lesion count, lesion SUV and functional tumor volume between [^68^Ga]Ga-DOTATOC and [^68^Ga]Ga-DOTA-TATE PET at any time point in an intrapatient comparison [41], supporting our view that also after therapeutic labeling we would not expect clinically relevant differences. Third, one patient with a G3 NET and a proliferation index Ki-67 of 23% was included because selected patients with G3 tumors may benefit from PRRT [3]. In four patients, neither Ki-67 nor grading was available which is in line with previous studies and represents a real-life clinical cohort [3]. Although Fisher’s exact test identified a higher frequency of responders in patients with ileum NETs and a lower frequency of responders in patients with unknown grading on a per-group basis, both parameters were not associated with PFS in cox regression analysis. Moreover, there were different reasons for discontinuation of PRRT. Although a more homogeneous cohort would have been desirable, similar reasons for discontinuation have been reported in other studies [1]. Finally, additional analyses of inflammatory pathways including cytokine profiling may provide important information. We consider this first work as a stimulus for more expansive efforts in the future. Furthermore, prospective trials are desired investigating modulation of the inflammatory response to ultimately improve patient outcomes.

## 5. Conclusions

Tumor-driven systemic inflammation may be associated with treatment response, change in tumor burden and prognosis in patients with GEP-NETs receiving PRRT. This study provides a rationale for further studies evaluating the role of tumor inflammation in targeted radionuclide therapy.

## Figures and Tables

**Table 1:** Characteristics of study population (*n* = 33).

Parameter	Total	Responders (*n* = 17)	Non-Responders (*n* = 16)	*p* Value
Sex no. (%)				0.49
Male	14 (42.4)	6 (35.3)	8 (50)	
Female	19 (57.6)	11 (64.7)	8 (50)	
Age (y)	63.4 ± 11.6	64.1 ± 11.2	62.6 ± 12.2	0.73
Body-mass index	25.7 ± 5.4	26.2 ± 6.9	25.1 ± 3.1	0.85
Previous therapies no. (%)				
Surgery	21 (63.6)	12 (70.6)	9 (56.3)	0.48
Chemotherapy	5(15.2)	2 (11.8)	3 (18.8)	0.66
Everolimus	5 (15.2)	2 (11.8)	3 (18.8)	0.66
None	8 (24.2)	3 (17.9)	5 (31.3)	0.44
Primary tumor site no. (%)				
Pancreas	10 (30.3)	4 (23.5)	6 (37.5)	0.46
Ileum	9 (27.3)	8 (47.1)	1 (6.3)	0.02
Small intestine, not otherwise specified	6 (18.2)	4 (23.5)	2 (12.5)	0.66
Rectum	2(6.1)	0	2 (12.5)	0.22
Colon, not otherwise specified	3 (9.1)	1 (5.9)	2 (12.5)	0.6
Unknown	3 (9.1)	0	3 (18.8)	0.1
Site of metastasis no. (%)				
Liver	31 (93.9)	17 (100)	14 (87.5)	0.23
Lymph nodes	24 (72.7)	13 (76.5)	11 (68.8)	0.71
Bone	20 (60.6)	9 (52.9)	11 (68.8)	0.48
Other	16 (48.5)	8 (47.1)	8 (50)	1
Ki-67 index no. (%)				
≤2% (G1)	7 (21.2)	6 (35.3)	1 (6.3)	0.09
3–20% (G2)	21 (63.6)	11 (64.7)	10 (62.5)	1.00
>20% (G3)	1 (3)	0	1 (6.3)	0.48
Unknown	4 (12.1)	0	4 (25.0)	0.045
Karnofsky index no. (%)				
≤70%	0 (0)	0	0	1
>70%	33 (100)	17 (100)	16 (100)	
Krenning score no. (%)				
Grade 2	0 (0)	0 (0)	0 (0)	1
Grade 3	16 (48.5)	8 (47.1)	8 (50)	
Grade 4	17 (51.5)	9 (52.9)	8 (50)	

Values in parentheses are percentages.

**Table 2:** Assessment of prognostic value of blood-based parameters for response prediction.

	Total Study Population		Responders (*n* = 17)		Non-Responders (*n* = 16)		*p* Value
	Mean ± SD	Range	Mean ± SD	Range	Mean ± SD	Range	
Erythrocytes (10^12^/L)	4.4 ± 0.6	3.1–5.4	4.6 ± 0.5	4–5.4	4.1 ± 0.6	3.1–5.2	0.05
Hemoglobin (g/dL)	12.4 ± 1.4	9–15.1	12.9 ± 0.9	11.7–15.1	11.9 ± 1.6	9–14.3	0.182
White blood cell count (10^9^/L)	6.9 ± 1.8	3.9–10.3	6.5 ± 1.2	3.9–8.6	7.4 ± 2.2	4.2–10.3	0.28
Absolute neutrophil count (10^9^/L)	4.6 ± 1.5	2.4–8.3	4.1 ± 0.8	2.4–5.3	7.3 ± 0.8	6.8–8.3	0.002
Absolute lymphocytes count (10^9^/L)	1.7 ± 0.7	0.6–2.8	1.7 ± 0.6	0.6–2.8	1.6 ± 0.8	0.8–2.3	0.871
Thrombocytes (10^9^/L)	264.4 ± 95.8	131–623	232.6 ± 45.7	142–298	483.8 ± 760.9	131–623	0.094
Thrombocyte volume (fL)	10.5 ± 0.8	9–12	10.6 ± 0.8	9.1–12	10.2 ± 0.9	9–12	0.169
CRP (mg/L)	10.3 ± 16.2	0.08–62.8	3 ± 4.4	0.08–15.4	18.1 ± 20.4	0.9–62.8	<0.001
ALP (U/L)	143 ± 128.6	31–714	110.5 ± 82.8	53–368	177.6 ± 159.7	31–714	0.045
PLR	178 ± 89.4	75.7–375	158.6 ± 71.3	75.7–316.4	250.7 ± 123.6	79.6–375	0.262
NLR	3.4 ± 2.1	1–8.5	2.8 ± 1.1	1–4.7	6.6 ± 3.1	3–8.5	0.056
PCM	3799 ± 7650	18–39,124	776 ± 1241	18–4451	7011 ± 10,092	254–39,124	<0.001
CRP-Albumin ratio	0.2 ± 0.5	0–2.1	0.079 ± 0.115	0–0.4	0.46 ± 0.63	0.02–2.1	0.01
SSR-TV (cm^3^)	170.2 ± 192.1	12.1–797.3	105.7 ± 123.7	12.1–495.8	238.6 ± 229.6	12.4–797.3	0.068
TL-SSR (cm^3^)	3283.3 ± 4065.2	60.8–15,868	2095.1 ± 2250.4	139.1–8372.2	4545.7 ± 5153.1	60.8–15,868	0.26

ALP: Alkaline phosphatase; CRP: C-reactive protein; NLR: Neutrophil-lymphocyte ratio; PCM: Platelet × CRP multiplier; PLR: Platelet-lymphocyte ratio; SD: Standard deviation; SSR-TV: Somatostatin receptor-derived tumor volume; and TL-SSR: Total lesion somatostatin receptor expression.

**Table 3:** Predictors of progression-free survival.

	Univariate Analysis			Multivariate Analysis		
	Hazard Ratio	95% CI	*p* Value	Hazard Ratio	95% CI	*p* Value
White blood cell count ≤ 6.5 × 10^9^/L	3.26	1.12; 9.44	0.03	20.26	1.91; 214.36	0.012
Absolute lymphocytes count ≤ 1.6 × 10^9^/L	0.99	0.14; 7.09	0.998			
Thrombocytes ≤ 260 × 10^9^/L	2.64	0.89; 7.81	0.079			
Thrombocyte volume ≤ 10.4 fL	1.05	0.32; 3.46	0.935			
C-reactive protein ≤ 2.5 mg/L	4.52	1.27; 16.18	0.02	226.325	3.47; 14768.04	0.011
CRP-Albumin ratio ≤ 0.0578	2.68	0.71; 10.17	0.146			
Platelet × CRP multiplier ≤ 910.45	3.98	1.25; 12.68	0.019	0.03	0.001; 1.3	0.068
Platelet-lymphocyte ratio ≤ 152.78	3.8	0.4; 36.58	0.248			
Neutrophil-lymphocyte ratio ≤ 2.87	0.86	0.29; 2.5	0.78			
Chromogranin A ≤ 386 μg/L	9.99	1.22; 81.93	0.032	7.009	0.57; 86.89	0.13
SSR-TV ≤ 78.87 cm^3^	5.12	1.41; 18.59	0.013	26.667	1.77; 401.25	0.018
TL-SSR ≤ 1590.98 cm^3^	3.02	0.97; 9.36	0.056			
Tumor origin (ileum vs other)	3.06	0.39; 24.11	0.29			
Tumor grading (G1-3 vs unknown)	1.83	0.54; 6.17	0.33			

CI: Confidence interval; CRP: C-reactive protein; SSR TV: Somatostatin receptor-derived tumor volume; and TL-SSR: Total lesion somatostatin receptor expression.

## Data Availability

The data are not publicly available because, due to the European regulations regarding data protection, we cannot make data available online or send it. However, all data are available for revision on-site.

## Supplementary Materials

The following are available online at https://www.mdpi.com/2075-4418/11/3/504/s1, Table S1: Predictors of change in tumor burden following two cycles of PRRT.
