# Supplementary material for: Predictive and Prognostic Impact of Blood-Based Inflammatory Biomarkers in Patients with Gastroenteropancreatic Neuroendocrine Tumors Commencing Peptide Receptor Radionuclide Therapy
Source: Diagnostics (Basel). 2021 Mar 12;11(3):504. doi: 10.3390/diagnostics11030504 (PMC8000284; doi:10.3390/diagnostics11030504)
Supplement: Supplementary file 1 [file diagnostics-11-00504-s001.pdf]

Predictive and prognostic impact of blood-based inflammatory biomarkers in patients with gastroenteropancreatic neuroendocrine tumors commencing peptide receptor radionuclide therapy

Ohlendorf et al.

Supplementary Material

Supplementary Table: 1

Supplementary Table 1. Predictors of change in tumor burden following 2 cycles of PRRT

	Univariate regression analysis			Multivariate regression analysis		
	Slope	95% CI	*P* value	Estimate	95% CI	P value
Image-derived parameters						
MTV	0.01361	-0.08123 to 0.1085	0.7717	-	-	-
TLG	0.0009700	-0.003505 to 0.005445	0.6615	-	-	-
Krenning grade	7.407	-13.07 to 27.89	0.4663	-	-	-
Lab values and inflammation indices						
Erythrocyte count	-10.47	-42.34 to 21.42	0.5081	-	-	-
Thrombocyte count	0.1493	-0.03311 to 0.3317	0.1051	-	-	-
Leukocyte count	13.81	4.954 to 22.67	0.0033	9.239	-32.04 to 50.51	0.6346
Absolute neutrophile count (ANC)	17.17	3.170 to 31.18	0.0193	-21.67	-80.34 to 36.99	0.4365
C-reactive protein (CRP)	0.6848	-0.4116 to 1.781	0.2122	1.474	0.3318 to 2.615	0.0157
Aspartate transaminase (AST)	-0.3557	-1.478 to 0.7669	0.5229	-	-	-
Alanine transaminase (ALT)	-0.03792	-0.5434 to 0.4675	0.8794	-	-	-
Gamma-glutamyltransferase (GGT)	0.005529	-0.1047 to 0.1158	0.9192	-	-	-
Lactate dehydrogenase (LDH)	0.1581	-0.1823 to 0.4984	0.3501	-	-	-
Cholinesterase (CHE)	0.2379	-3.416 to 3.892	0.8927	-	-	-
Albumine	1.142	-2.930 to 5.214	0.5692	-	-	-
Alkaline phosphatase (ALP)	0.03093	-0.1105 to 0.1724	0.6588	-	-	-
Chromogranin A (CgA)	0.003255	-0.0008655 to 0.007375	0.1161	-	-	-
Platelet x CRP multiplier (PCM)	0.001422	-0.0009055 to 0.003750	0.2220	-		
Platelet-lymphocyte ratio (PLR)	0.2491	0.01981 to 0.4784	0.0349	-0.2965	-0.6072 to 0.01408	0.0596
Neutrophil-lymphocyte ratio (NLR)	15.87	7.660 to 24.09	0.0008	30.39	11.70 to 49.08	0.0040
CRP-albumin ratio (CRP/Alb ratio)	-1.314	-38.26 to 35.63	0.9423	-	-	-
High-sensitivity inflammation-based prognostic index (HSPI)	2.046	-34.04 to 38.13	0.9087	-	-	-
